# Data on the synthesis and characterizations of carboxylated carbon-based catalyst from eucalyptus as efficient and reusable catalysts for hydrolysis of eucalyptus

**DOI:** 10.1016/j.dib.2020.105520

**Published:** 2020-04-17

**Authors:** Hassan Idris Abdu, Kamel Eid, Aboubakr M. Abdullah, Xiaoquan Lu

**Affiliations:** a Key Laboratory of Bioelectrochemistry & Environmental Analysis of Gansu Province, College of Chemistry & Chemical Engineering, Northwest Normal University, Lanzhou 730070, China; b Centre for Advanced Materials, Qatar University, Doha 2713, Qatar; c Tianjin Key Laboratory of Molecular Optoelectronic Sciences, Department of Chemistry, Tianjin University, Tianjin 300072, China

**Keywords:** Eucalyptus, Biomass, Biofuel, Carboxylated carbon catalyst, Heterogeneous catalysts

## Abstract

The presented article reports the preparation and characterization of heterogeneous carbon catalyst enriched with carboxylic group denoted as (ECS) from *Eucalyptus* as an efficient catalyst for the hydrolysis of woody *Eucalyptus biomass*. The fabrication process is based on the ball milling of *Eucalyptus* as a carbon source in the presence of dry ice as an oxidizing agent followed by acidification with the assistance of hydrochloric acid. The data are including the schematic for the full synthesis steps and characterization tools in addition to the thermogravimetric analysis and proton nuclear magnetic resonance analysis for the ECS catalyst. Meanwhile, the catalytic performance of ECS catalyst towards the hydrolysis of *Eucalyptus* was measured under different temperatures ranged from 160  to 200 °C. The ECS catalyst allowed the selective hydrolysis of *Eucalyptus* to glucose and xylose, as proved by high-performance liquid chromatography. The data herein are associated with the article entitled “ Unveiling one-pot fabrication of scalable and reusable carboxylated heterogeneous carbon-based catalyst from *Eucalyptus* plant with the assistance of dry Ice for selective hydrolysis of *Eucalyptus* Biomass’’ [1].

Specifications table

**Table:** 

Subject	Chemistry
Specific subject area	General chemistry, including developing catalysis for hydrolysis of *Eucalyptus* to xylose and glucose fuel.
Type of data	Image Chart Graph Figure
How data were acquired	Thermogravimetric analysis (TGA) was recorded on (TGA, STA 449 F3, NETZSC, Germany). Proton nuclear magnetic resonance (^1^H NMR) spectra were measured by BrukerAvance III 400 MHz spectrometer equipped with a 5 mm SEI probe with a z-gradient (Bruker, Rheinstetten, Germany). The high-performance liquid chromatography ((HPLC), Agilent 1260 series, USA) with an Aminex HPX-87H column (300 × 7.8 mm, Bio-Rad, USA). Photographs for the Ball miller system were Canon DSLR Camera EOS 80D 18 mm Lens and collected using Microsoft PowerPoint. All the charts for TGA, HPLC, and ^1^H NMR were drawn using OriginPro 2018.
Data format	Raw data TGA, HPLC, and ^1^H NMR
Parameters for data collection	The hydrolysis process was carried out under different temperatures, including 160 °C, 180 °C, and 200 °C.
Description of data collection	The preparation steps, analysis apertures, thermal stability, and chemical structure of the as-obtained catalysts. The hydrolysis of Eucalyptus biomass was carried out at different reaction temperatures along with the identification of the products of the hydrolysis using HPLC.
Data source location	Key Laboratory of Bioelectrochemistry & Environmental Analysis of Gansu Province, College of Chemistry & Chemical Engineering, Northwest Normal University, Lanzhou 730070, P. R. China Center for advanced materials, Qatar University, Doha 2713, Qatar Tianjin Key Laboratory of Molecular Optoelectronic Science, Department of Chemistry, School of Science, Tianjin University, Tianjin, 300072, P. R. China
Data accessibility	The data are provided herein in this article. All the raw data were provided as a supplementary Microsoft Excel file contains the raw data for TGA, ^1^H NMR, and hydrolysis process.
Related research article	Abdu, H.I., et al. Unveiling one-pot fabrication of scalable and reusable carboxylated heterogeneous carbon-based catalyst from *Eucalyptus* plant with the assistance of dry ice for selective hydrolysis of *Eucalyptus* Biomass. [1] https://doi.org/10.1016/j.renene.2020.02.034

## Value of the data

• Controlling the preparation and characterizations of heterogeneous carbon-based catalysts is vital in the biofuel application.
• The carboxylated carbon catalyst was prepared from *Eucalyptus* and used for the hydrolysis of woody *Eucalyptus biomass.*
• The catalytic hydrolysis *Eucalyptus* was benchmarked at different temperatures to understand the reaction kinetics.
• The as-obtained catalyst can be easily recycled and reused after the hydrolysis process.
• The presented data are essential for the preparation of reusable heterogeneous catalyst from various plants.

## 1 Data description

**Scheme 1:**
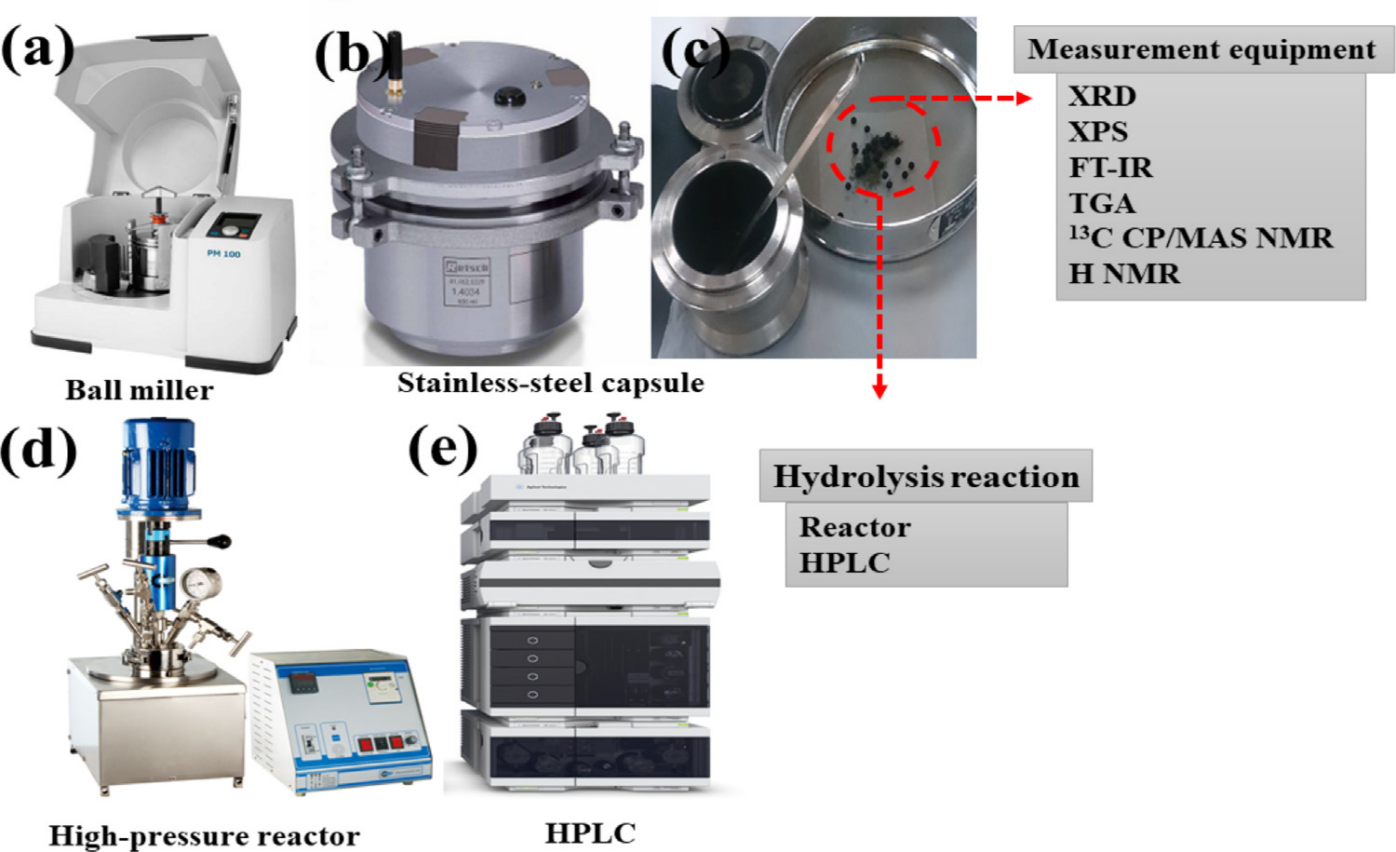
Illustrates the detailed schematic of the experimental apparatus and measurement equipment.

**Fig. 1:**
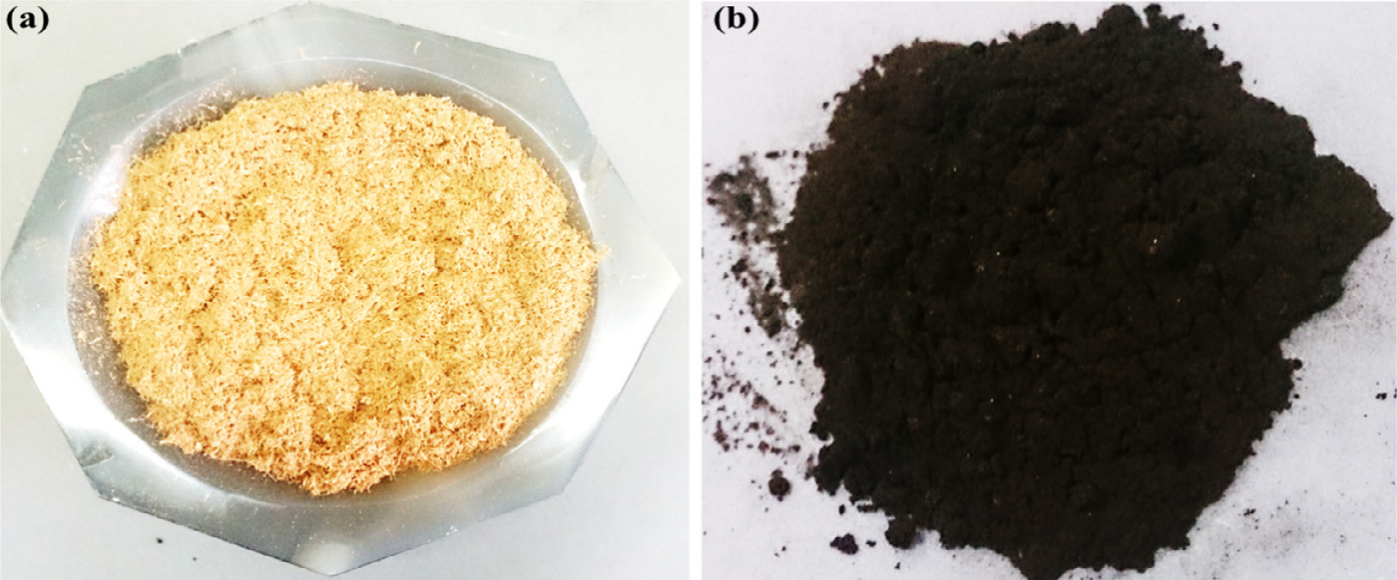
Photographs of (a) *Eucalyptus* and (b) ECS catalyst.

**Fig. 2:**
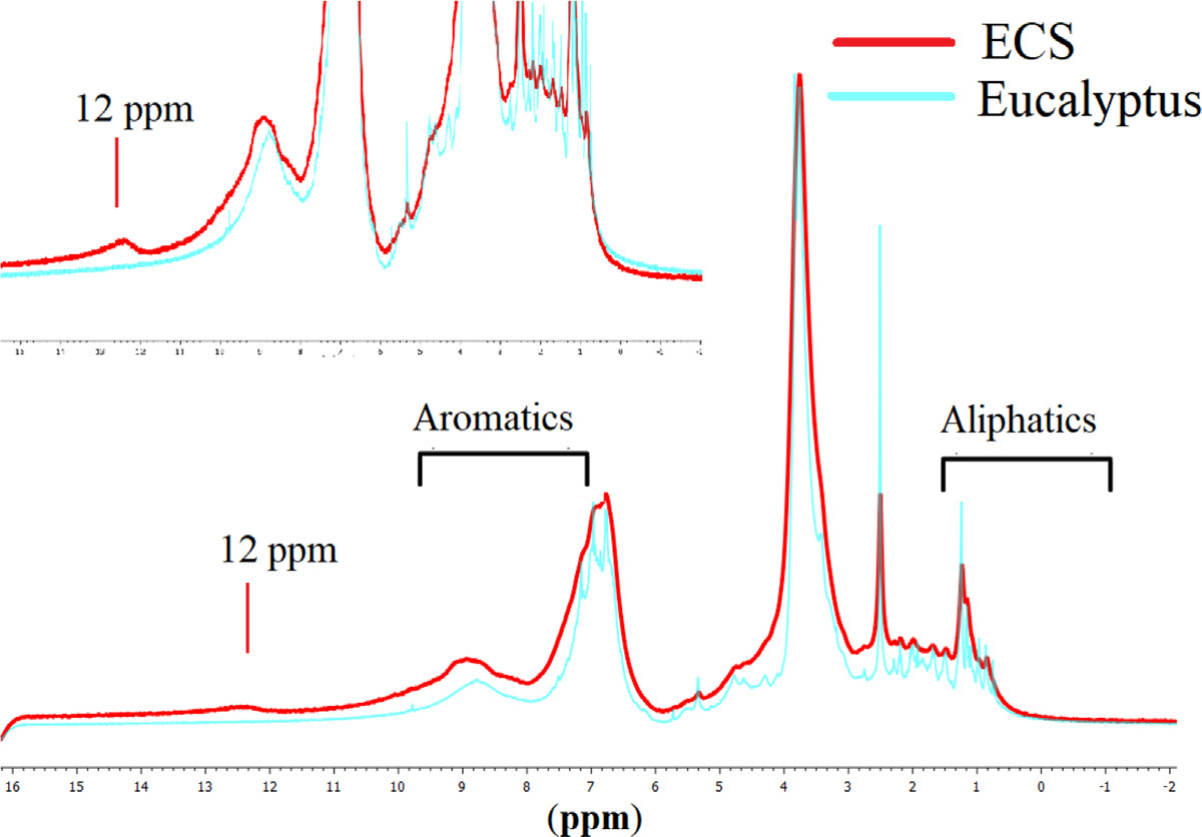
The ^1^H NMR spectrum of ECS and pristine Eucalyptus. The insight shows the magnification of the area between 16  and 7 ppm for both ECS catalyst and *Eucalyptus* to confirm the formation of COOH group inside ECS catalyst.

**Fig. 3:**
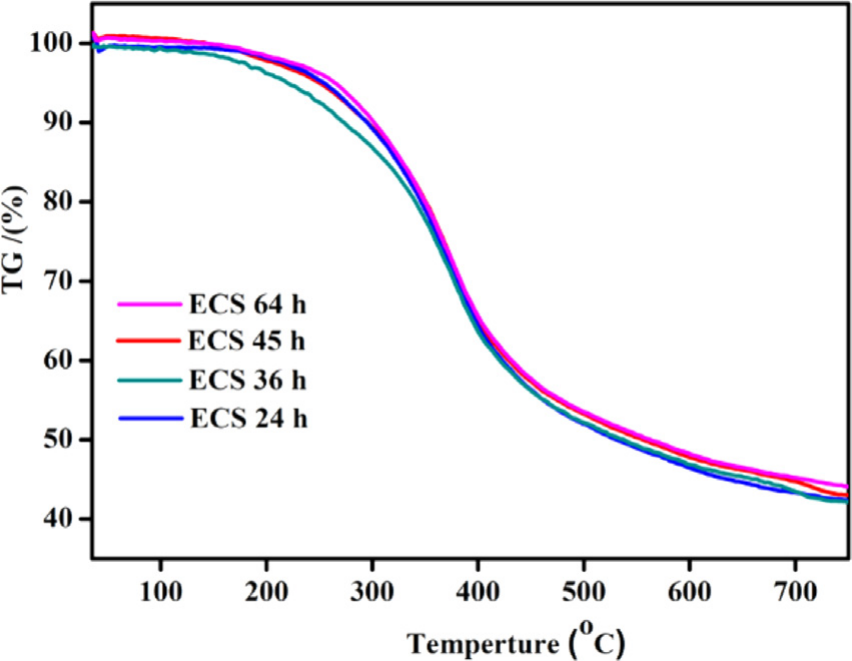
The TGA for *ECS* catalysts prepared under different milling times.

**Fig. 4:**
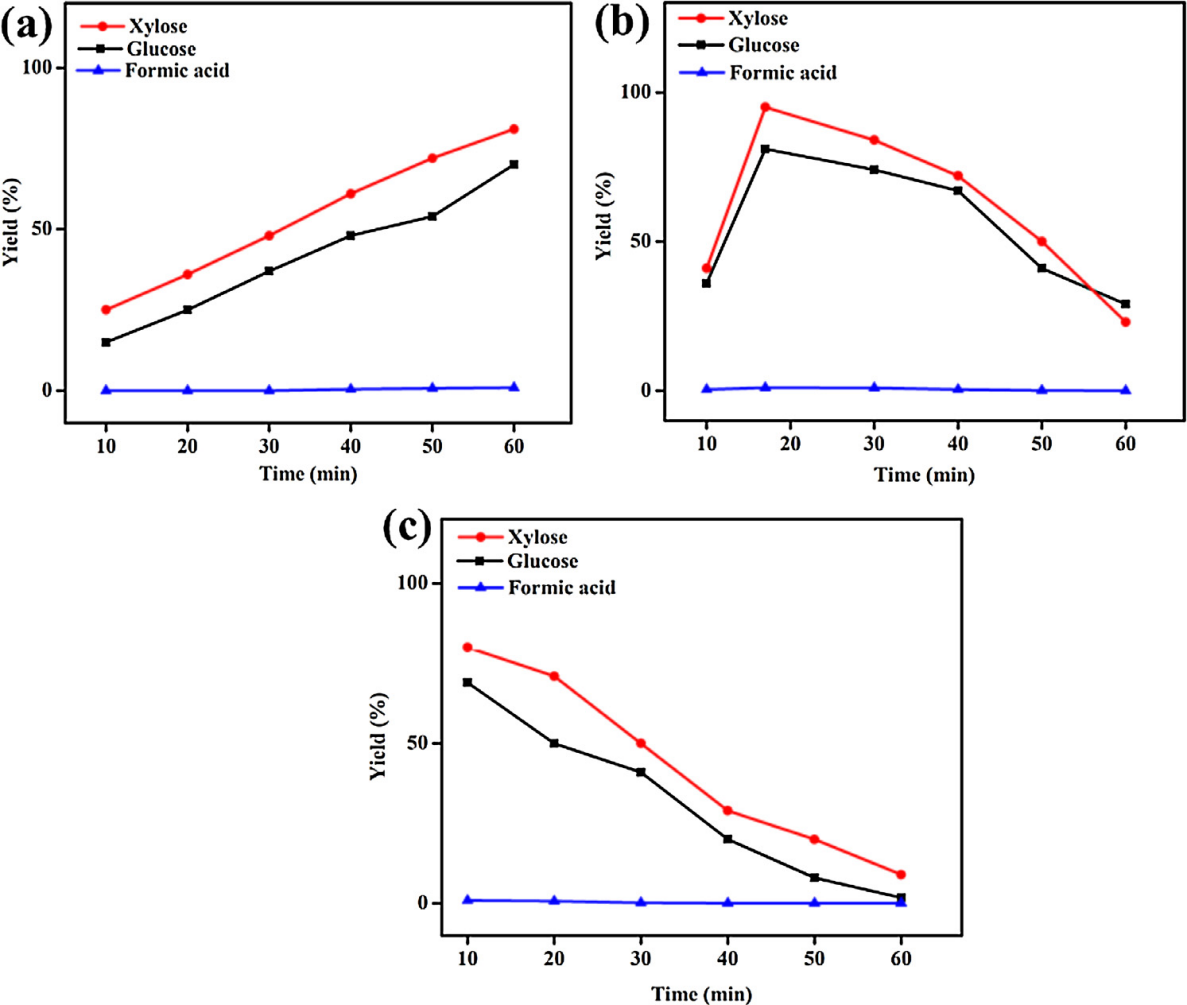
Hydrolysis of *Eucalyptus* by *ECS* catalysts under different temperatures and times for (a) at 160 , (b) 180  and (c) 200 °C.

The data presented in this article are related to the rational design of efficient and reusable ECS catalysts from *Eucalyptus* plant and its utilization for the hydrolysis of *Eucalyptus* plant [1]. The data including the detailed schematic of the experimental, characterization, and hydrolysis application apparatus, including ball-miller for the preparation of ECS catalyst from *Eucalyptus*, high-pressure rector for measuring the catalytic performance over *Eucalyptus* plant, and HPLC for determination the hydrolysis products as well as characterization tools (Scheme 1). The photographs for showing the difference between the pristine *Eucalyptus* plant and the ECS catalyst (Fig. 1). This is in addition to using the ^1^H NMR for identifying the chemical structure of the as-obtained ECS catalysts compared to natural *Eucalyptus* plant (Fig. 2) and the TGA to investigation the thermal stability of ECS catalysts obtained at different milling times including 24, 36, 45, and 64 h (Fig. 3). Then, the hydrolysis products of *Eucalyptus* on the as-synthesized ECS catalyst carried out under different temperatures, including 160  (Fig. 4a), 180  (Fig. 4b) and 200 °C (Fig. 4a) as well as the hydrolysis products obtained during the reaction times (Fig. 4).

## 2 Experimental design, materials, and methods

### 2.1 Preparation process of ECS

Scheme S1 shows a schematic of the experimental and characterization apparatus for the formation of ECS catalyst from woody *Eucalyptus* biomass [1,2]. The preparation process, including mixing *Eucalyptus* and dry ice in a stainless-steel pot (250 ml) contains stainless-steel balls (5 mm), and then milling at 550 rpm using Ball miller (Fritsch P-6 planetary) for 2 h, followed by filtration, washing, and finally drying under air. The content of the carboxylic (COOH) group inside ECS catalyst could be easily controlled by adjusting the milling time. The obtained ECS catalysts were confirmed using various characterization tools such as TGA for thermal stabilities and ^1^H NMR for chemical structure [1,^3^,4]. The high-pressure reactor for the hydrolysis process and HPLC for the identification of the products.

Fig. 1 shows the photographs of pure *Eucalyptus* and the obtained ECS, which indicates changing the color of pure *Eucalyptus* from the pale brown to deep black color after the carboxylation process and annealing (Fig. S1).

Fig. 2 shows the ^1^H NMR spectra for both ECS catalyst and pure Eucalyptus, which both showed the signals attributed to the H of the aliphatic carbons between 0.9 and 1.6 ppm in addition to the signals between 6.3 and 9.5 ppm [5], [6], [7]. Meanwhile, the ECS catalyst reveals the signal assigned to H bonded to the C = O group at 12.0 ppm, indicating the successful formation of ECS catalyst composed of C-aromatic skeletal enriched with COOH group and some aliphatic moieties [5], [6], [7]. The ^1^H NMR displayed the absence of undesired products, except the signal at 2.5 and 3.35 ppm originated from H of *dimethyl sulfoxide* solvent.

The thermal stability of the as-synthesized ECS catalyst prepared under different milling times, comprising 24, 36, 45, 64 h, were investigated by the TGA, as shown in Fig. 3. There is no any kind of noticed significant changes among all prepared ECS catalysts [8]. Meanwhile, all ECS catalysts were thermally stable until 200 °C (Fig. S3). The major decompositions of ECS were achieved between 250 and 450 °C as the carbon-based catalysts are highly stable thermally and chemically [9].

Fig. 4 depicts the catalytic performance of ECS catalyst over *Eucalyptus* benchmarked at 160, 180, and 200 °C in the presence of 120 ppm of HCl, and the reaction products were estimated using the HPLC at 10 min intervals each. It should be noticed that, before any hydrolysis process, both *Eucalyptus* and *ECS* were milled together for 2 h to enhance the contact between them in line with elsewhere reports [10], [11], [12], [13], [14]. In particular, at 160 °C, the hydrolysis of *Eucalyptus* increased with time till achieving the maximum glucose yield of (70%) and xylose yield of (82%) after 1 h (Fig. 4a). Likewise, at 180 °C, the hydrolysis activity increased quickly until 17 min to reach the maximum production yield of (81%) for glucose and (95%) of xylose, followed by quickly decreasing until reaching the lowest yield of nearly (10%) for xylose and (15%) for glucose after 1 h (Fig. 4b). At 200 °C, the hydrolysis enhanced promptly to reach the maximum value of glucose (69%), and xylose (80%) after 10 min and then decreased quickly (Fig. 4c). Under all the temperatures used, only an inferior amount of furfural acid was detected. All data showed that the kinetics of hydrolysis at 200 °C was higher than that at 180 °C and 160 °C, respectively [15]. Meanwhile, the optimum reaction temperature was 180 °C.

## Appendix Supplementary material

Image, application 1
